# Antiandrogen Withdrawal Syndrome After Discontinuation of Enzalutamide in Patients With Metastatic Castration-Resistant Prostate Cancer: A Report of Two Clinical Cases and a Literature Review

**DOI:** 10.7759/cureus.63506

**Published:** 2024-06-30

**Authors:** Patrícia R Rodrigues, Cátia Faustino, Joaquina Maurício, Filipa Carneiro

**Affiliations:** 1 Medical Oncology, Instituto Português de Oncologia do Porto Francisco Gentil, Centro Hospitalar de Lisboa Central (EPE), Porto, PRT

**Keywords:** aaws, enzalutamide withdrawal syndrome, antiandrogen withdrawal syndrome, castration-resistant metastatic prostate cancer, enzalutamide

## Abstract

Metastatic prostate cancer treatment is based on androgen deprivation, with pharmacological or surgical castration. This treatment may be complemented with the addition of antiandrogenic drugs. In the setting of prostate-specific antigen (PSA) progression and subsequent suspension of the antiandrogenic drug, there might occur a phenomenon of antiandrogen withdrawal, leading to a decrease in PSA and/or improvement in imaging or clinical outcomes after discontinuation of the antiandrogenic agent. Although there are some descriptions of withdrawal after the cessation of enzalutamide, the physiological mechanism behind it, as well as its frequency and impact on patient survival, remain unknown. We present two clinical cases of antiandrogenic withdrawal after enzalutamide discontinuation and discuss potential contributing factors to this phenomenon.

## Introduction

Prostate cancer is the second most common cancer worldwide, with more than 1.4 million new cases globally in 2020 [1]. In Portugal, 6,912 new cases were recorded in 2019 (data from the national cancer registry of 2019) with a standardized incidence rate for the European population of 90.9 per 100,000 inhabitants. Its prevalence is increasing due to population growth and aging and to increasing overall survival (OS) of these patients, probably related to new and more effective treatment options [2]. This cancer is unique because of its relationship with androgens as a growth factor. Hence, androgen deprivation is the major strategy in clinical practice, and metastatic prostate cancer is divided into two clinical states regarding its sensitivity to androgen deprivation therapy (ADT) as “hormone-sensitive prostate cancer” (mHSPC) and “castration-resistant prostate cancer” (mCRPC) [3].

In the metastatic setting, prostate-specific antigen (PSA) levels are used for monitoring treatment response, and a rise in PSA levels often indicates disease progression before radiological progression, although up to a quarter of patients may experience disease progression without an increase in PSA levels. On the other hand, a decrease in PSA levels is usually correlated with disease control [4-6].

Antiandrogen withdrawal syndrome (AAWS) is defined as a decrease in PSA levels in more than half of the baseline value after discontinuation of antiandrogen therapy in the context of combined androgen blockade [7]. This was initially described for older agents, such as flutamide, bicalutamide, and nilutamide; moreover, before the approval of enzalutamide and abiraterone, this phenomenon could be used to postpone the beginning of chemotherapy in mCRPC [7]. AAWS after enzalutamide is not well established. Some evidence suggests that AAWS may happen with enzalutamide in a minority of patients with mCRPC. We report two cases in which AAWS after enzalutamide occurred.

## Case presentation

Case 1

A 68-year-old male, Eastern Cooperative Oncology Group performance status (ECOG PS) of 0, with a medical history of hypertension, dyslipidemia, and benign prostate hyperplasia, was diagnosed in June 2013 with prostate cancer Gleason 9 (4+5). He was submitted to radical prostatectomy, and the tumor was staged as pT3a, pN0, cM0, and R0 surgery, with a detectable PSA after surgery (0.45 ng/mL). He underwent postoperative radiotherapy in December 2013 (external radiotherapy to the pelvic region, total radiation of 66 Gy in 33 fractions). The PSA nadir was 0.229 ng/mL (four months after the end of radiotherapy).

**Figure 1:**
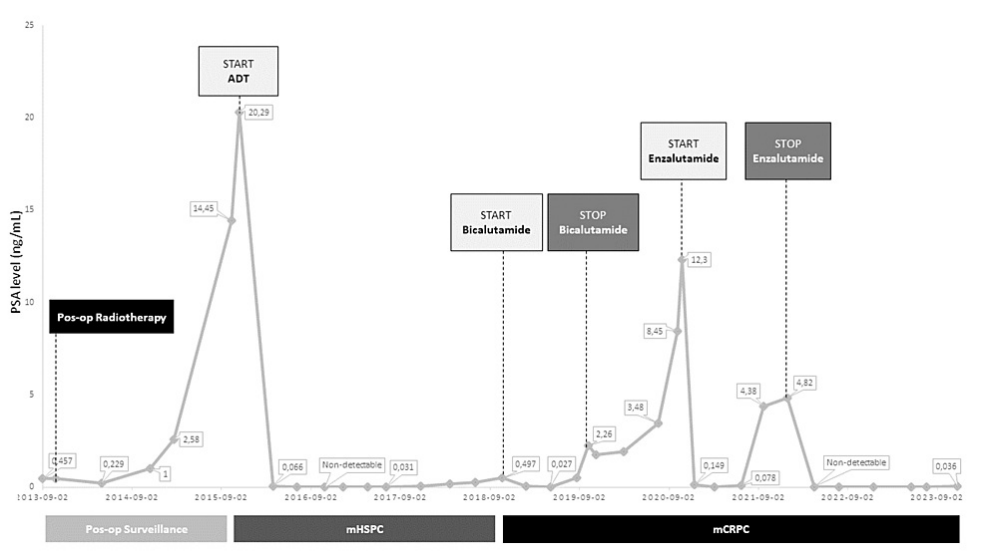
Schematic representation of the evolution of PSA over time for Case 1, since radical prostatectomy, with the introduction and cessation of indicated interventions and drugs. ADT: androgen deprivation therapy; mCRPC: metastatic castration-resistant prostate cancer; mHSPC: metastatic hormone-sensitive prostate cancer; post-op: postoperative; PSA: prostate-specific antigen. Dates are presented as year-month-day.

Two years after the diagnosis, biochemical recurrence occurred (PSA 14.45 ng/mL) with radiological evidence of bone and nodal metastases, defining mHSPC. The patient was asymptomatic and in December 2015, ADT was started with three-monthly goserelin 10.8 mg subcutaneously. Figure 1 shows a schematic representation of the evolution of PSA over time and the drugs administered.

After 34 months of treatment, there was the emergence of new nodal metastases, indicating castration-resistant prostate cancer (according to Response Evaluation Criteria in Solid Tumors (RECIST) 1.1 criteria) [8]. Bicalutamide was associated, and nadir was achieved seven months later (0.027 ng/mL).

In October 2019, because of biochemical progression, bicalutamide was stopped. The patient still had stable radiological disease without clinical progression and maintained ADT monotherapy. A year later, the patient presented with intermittent lower back pain. It was documented at that time radiological progression by RECIST 1.1 and PCWG3 criteria, with new bone and nodal metastases. As the patient presented with few symptoms, enzalutamide and zoledronic acid were started. During the treatment, the patient maintained lower back pain, which resolved with paracetamol on an as-needed basis, and reported asthenia graded as 1 as per the Common Terminology Criteria for Adverse Events (CTCAE).

In January 2022, the patient presented radiological disease progression and increasing PSA levels (4.82 ng/mL), leading to the discontinuation of enzalutamide. The PSA value three months later had decreased by almost 100% (nonmeasurable value), and so it remained until May 2023, when it was detectable (at a value of 0.01 ng/mL). The last PSA record was in November 2023, with a value of 0.036 ng/mL. To date, the patient maintains ADT (with goserelin) and zoledronic acid, with disease control, being paucisymptomatic without radiological progression.

Case 2

**Figure 2:**
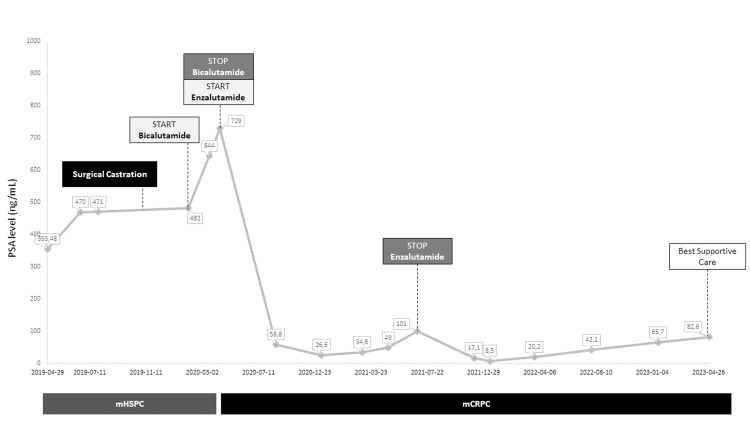
Schematic representation of the evolution of PSA over time for Case 2, since the initial evaluation and diagnosis, with the introduction and cessation of indicated interventions and drugs. mCRPC: metastatic castration-resistant prostate cancer; mHSPC: metastatic hormone-sensitive prostate cancer; PSA: prostate-specific antigen. Dates are presented as year-month-day.

An 80-year-old male, ECOG PS 0, with a medical history of long-standing medicated hypertension without known complications, presented in April 2019 with lower urinary tract symptoms (LUTS). Laboratory data showed a total PSA of 355.48 ng/mL. Digital rectal examination revealed a stone-like prostate gland, adherent to surrounding tissues. A prostatic biopsy performed in August 2019 revealed a Gleason 9 (4+5) prostate adenocarcinoma. A CT scan showed pelvic and retroperitoneal enlarged lymph nodes. Bone scintigraphy was negative for osteoblastic bone metastases. Therefore, the disease was staged as cT4 N+ M1 hormone-sensitive prostate cancer. Figure 2 shows the PSA evolution over time and interventions.

He underwent surgical castration in November 2019. However, there was no PSA response (castration serum testosterone levels were confirmed). Bicalutamide 50 mg daily was added in March 2020, but PSA still increased after the combination, and although radiological evaluation showed stable disease, the patient’s symptoms worsened, with increasing LUTS and peripheral edema, meeting criteria for mCRPC. He was then proposed for treatment with docetaxel, which the patient refused. Enzalutamide 160 mg was started in May 2020, with a baseline PSA value of 729 ng/mL. There was a radiological partial response associated with a biochemical response (PSA nadir of 26.6 ng/mL) seven months later, associated with a clinical response (resolution of peripheral edema and LUTS improvement).

Over time, the PSA increased, and in July 2021, there was radiological confirmation of nodal disease progression. Enzalutamide was discontinued in August 2021, with a PSA value of 101 ng/mL. Once again, chemotherapy with docetaxel was proposed, but the patient still refused. An immunohistochemical assessment of the prostate biopsy was performed, which revealed a loss of MSH6 expression, indicating a microsatellite instability-high (MSI-H) phenotype. He was then proposed for immunotherapy with pembrolizumab, but the patient also refused this treatment.

In November 2021, PSA levels had a significant decrease and continued decreasing to a nadir of 8.5 ng/mL in December 2021 (a 92% reduction). A CT scan in March 2022 revealed stable disease according to RECIST 1.1. PSA levels started to increase in April 2022 (eight months since enzalutamide suspension), and, in March 2023, it was confirmed as disease progression. The patient and family maintained their refusal of treatment with docetaxel or pembrolizumab, so he was proposed for exclusive best supportive care.

## Discussion

The two cases presented showed a decrease in PSA levels after discontinuation of enzalutamide in patients with mCRPC, without adding a new drug component, confirming AAWS to enzalutamide. The first case shows an AAWS response duration of at least 19 months and is still ongoing, while the second case had four months of PSA sustained response associated with radiological response. Neither of these patients was exposed to chemotherapy previously, and both had been treated with bicalutamide before enzalutamide. Moreover, it is worth considering that one of the cases discussed presented MSI-H prostate cancer, and the relevance of this status to AAWS is unknown.

This phenomenon, although well-described for first-generation antiandrogens [7], is not well understood, and the incidence and characterization of AAWS for enzalutamide have not yet been well established, although there are some studies with a small cohort of patients to acknowledge this entity. A study performed by Rodriguez-Vida et al. [9] in the UK, which included 30 patients with mCRPC previously exposed to docetaxel and with disease progression on enzalutamide, showed one patient with a PSA decline of more than 50% after stopping enzalutamide and one patient with a PSA decline between 30% and 50%. The first patient was under enzalutamide for 21.4 months, while the second patient was for only 4.2 months. Neither had radiological evaluation after withdrawal. In this study, there was no difference between OS of the patients with AAWS and those without (but this result is limited by a small sample), and there was no symptomatic benefit. On the other hand, a retrospective study by von Klot et al. [10] evaluated 31 patients with mCRPC, and none had a decreased PSA after enzalutamide withdrawal. A study by Poole et al. [11] included 47 patients with mCRPC with PSA progression under enzalutamide, in which one patient had a documented AAWS with a PSA decline of 87%, and four patients had a PSA decline inferior to 50%. The median AAWS response duration was 3.3 months.

The mechanism underlying AAWS is not clear, but theories include alterations in the androgen receptor (AR). The AR has a key role in all the phases of prostate cancer [7]. It is a nuclear protein with four different domains: the N-terminal transactivation domain (NTD), the DNA-binding domain, a hinge region, and the ligand-binding domain (LBD). Usually, dihydrotestosterone (DHT) and testosterone are the only molecules that bind to LBD [7]. It is believed that progression under ADT is because of the ability of cancer cells to overcome a low androgen environment through upregulation of enzymes involved in androgen synthesis, producing androgen and stimulating their own growth, or overexpression of the AR or mutations of the AR gene [7].

Enzalutamide is a second-generation non-steroidal antiandrogen that significantly improved OS and progression-free survival (PFS) in patients with metastatic castration-resistant prostate cancer (mCRPC) previously exposed to docetaxel [12] and in chemotherapy-naïve (PREVAIL study) patients [13] when used in combination with ADT. It is a potent oral AR inhibitor that does not require co-administration of corticosteroids. It inhibits the AR signaling in three ways: 1) potent competitive binder of the AR, 2) prevention of translocation of the AR from the cytoplasm to the nucleus, and 3) inhibition of the AR binding to chromosomal DNA, preventing gene transcription [14]. Thus, enzalutamide was designed to have only an antagonistic effect on the AR, without the potential for agonism. For this reason, theoretically, AAWS should not occur after enzalutamide, as the cell signaling pathways through the AR would be inhibited.

However, over the years, some studies on the mechanism of resistance to enzalutamide have revealed mutations in the LBD region of the AR, particularly the F876L missense mutation, which confers agonist activity to enzalutamide, both in vitro and in vivo [15]. Cells with this mutation are dependent on enzalutamide for cellular growth under androgen deprivation conditions [16]. Therefore, withdrawal in these patients may be beneficial. It is possible to determine the presence of this mutation in circulating tumor DNA (ctDNA) using the (beads, emulsions, amplification, and magnetics (BEAMing) method [15]. This could be useful in clinical practice to determine a subset of patients, for whom the withdrawal of enzalutamide could be beneficial, although it is very rare. In none of the mentioned studies was ctDNA collected to determine the existence of this mutation, and the same was not done in the two cases we presented either.

Nonetheless, there are no data to this day about the incidence of AAWS with enzalutamide, if it has an impact on OS, the choice of future treatments, or if it is related to the F876L mutation or other mechanisms of resistance to enzalutamide that overpass the AR.

## Conclusions

Despite the mechanism of action of enzalutamide as a pure antagonist, the occurrence of AAWS is possible upon its suspension. The frequency of this phenomenon is not yet known and could be a rare or unrecognized occurrence among clinicians. It is also unclear whether this phenomenon impacts the patient's OS by delaying the initiation of a subsequent line of treatment. Furthermore, it is unknown whether there is a connection with the emergence of the F876L mutation or other as-yet-unknown mechanisms. Further studies on this phenomenon are necessary to establish guidelines for when AAWS occurs after enzalutamide.
